# Bruton Tyrosine Kinase Inhibitor-Based Therapy Versus Rituximab-Containing Regimens for Chronic Lymphocytic Leukemia: A Systematic Review and Meta-Analysis of Randomized Controlled Trials

**DOI:** 10.7759/cureus.115923

**Published:** 2026-09-07

**Authors:** Sadia Yousaf, Maryam Akhtar, Uswah Ilyas, Maham Fatima, Arshbeer Singh Sandhu, Haris Mumtaz Malik, Tayyab Mumtaz Khan, Sana Mansoor, Muhammad Awais Bin Abdul Malik

**Affiliations:** 1 Internal Medicine, King Edward Medical University, Lahore, PAK; 2 Internal Medicine, Sir Ganga Ram Hospital, Lahore, PAK; 3 Neurosciences, University Hospital Coventry and Warwickshire, Coventry, GBR; 4 Internal Medicine, Rawalpindi Medical University and Allied Hospitals, Rawalpindi, PAK; 5 Orthopaedic Surgery, Rawalpindi Medical University, Rawalpindi, PAK; 6 Pediatrics, Rawalpindi Medical University, Rawalpindi, PAK; 7 Internal Medicine, AdventHealth, Orlando, USA

**Keywords:** acalabrutinib, bruton tyrosine kinase inhibitor, chronic lymphocytic leukemia, ibrutinib, meta-analysis, pirtobrutinib, rituximab, small lymphocytic lymphoma, systematic review, zanubrutinib

## Abstract

Bruton tyrosine kinase (BTK) inhibitors have increasingly replaced rituximab-based chemoimmunotherapy in chronic lymphocytic leukemia (CLL), but the magnitude of benefit across treatment settings and whether it extends to overall survival remains uncertain. Therefore, we compared BTK inhibitor-based therapy with rituximab-containing regimens in randomized controlled trials (RCTs). PubMed/MEDLINE and the Cochrane Central Register of Controlled Trials were searched from inception to August 2, 2026, for RCTs enrolling adults with CLL or small lymphocytic lymphoma that compared a BTK inhibitor-based strategy with a rituximab-containing comparator and reported progression-free survival (PFS), overall survival (OS), or overall response rate (ORR). Two reviewers independently screened records, extracted data, and assessed risk of bias using RoB 2. Hazard ratios (HRs) were pooled using the generic inverse-variance method and risk ratios (RRs) using the Mantel-Haenszel method, both under random-effects models; heterogeneity was quantified using I². The review followed PRISMA 2020. Eight RCTs comprising 2,781 participants were included, spanning treatment-naive, relapsed or refractory, and covalent BTK inhibitor-pretreated disease. Five trials were at low overall risk of bias, and three raised some concerns. BTK inhibitor-based therapy was associated with significantly longer PFS (eight trials; pooled HR, 0.42; 95% confidence interval (CI): 0.24-0.73; P=0.002), with substantial heterogeneity (I²=93%). Overall response occurred in 440 of 543 patients (81.0%) receiving BTK inhibitor-based therapy versus 350 of 491 (71.3%) receiving rituximab-containing regimens, a nonsignificant difference (four trials; RR: 1.18; 95% CI: 0.98-1.42; P=0.08; I²=86%). OS favored BTK inhibitor-based therapy without reaching statistical significance (six trials; HR: 0.67; 95% CI: 0.40-1.11; P=0.12; I²=48%). Publication bias could not be formally assessed because fewer than 10 trials contributed to any outcome. BTK inhibitor-based therapy was associated with significantly longer PFS compared with rituximab-containing regimens, whereas the evidence for OS and ORR remained inconclusive. Very high heterogeneity indicates that the pooled PFS estimate represents an average across clinically distinct treatment questions rather than a uniform class effect, and treatment selection should remain individualized.

## Introduction and background

Chronic lymphocytic leukemia (CLL) is the most common adult leukemia in Western countries. CLL is characterized by the clonal proliferation and accumulation of mature CD5-positive B lymphocytes within the peripheral blood, bone marrow, lymph nodes, and spleen [1]. Despite its generally indolent clinical course, CLL demonstrates considerable biological and clinical heterogeneity, with disease progression ranging from prolonged asymptomatic survival to rapidly progressive disease requiring therapeutic intervention. Current treatment recommendations are based on the International Workshop on Chronic Lymphocytic Leukemia (iwCLL) criteria and incorporate disease stage, patient age, performance status, comorbidities, and molecular and cytogenetic risk factors to guide individualized treatment decisions [2].

Rituximab-based chemoimmunotherapy historically represented a major treatment approach for CLL. Regimens such as fludarabine, cyclophosphamide, and rituximab (FCR), bendamustine plus rituximab (BR), and chlorambucil plus rituximab demonstrated clinically meaningful response rates and progression-free survival in selected treatment-naïve and relapsed or refractory populations [3,4]. However, these regimens are associated with substantial hematologic toxicity, prolonged immunosuppression, infectious complications, and limited efficacy in patients with high-risk genetic abnormalities such as TP53 disruption and del(17p) [1,2,5]. These limitations, together with frequent disease relapse following chemoimmunotherapy, contributed to the transition toward targeted therapeutic approaches.

Advances in the understanding of B-cell receptor signaling identified Bruton tyrosine kinase (BTK) as a key therapeutic target in CLL. BTK plays a pivotal role in regulating malignant B-cell proliferation, survival, migration, and interaction with the tumor microenvironment. Inhibition of BTK interrupts these signaling pathways, thereby suppressing leukemic cell survival and disease progression [1]. The introduction of the first-generation BTK inhibitor, ibrutinib, marked a major shift in CLL treatment after randomized clinical trials demonstrated superior efficacy compared with anti-CD20-based therapy in relapsed disease and with chlorambucil in the first-line setting [6,7]. Subsequently, second-generation BTK inhibitors, including acalabrutinib and zanubrutinib, were developed with greater kinase selectivity and the potential for reduced off-target toxicities, including cardiovascular and bleeding events [8-11]. More recently, newer BTK inhibitor-based strategies, including the non-covalent inhibitor pirtobrutinib, have further expanded treatment options for patients with treatment-naïve and relapsed or refractory CLL [12,13].

Several randomized controlled trials (RCTs) have evaluated BTK inhibitor-based strategies against rituximab-containing comparator regimens across different CLL treatment settings. The ASCEND trial demonstrated significantly prolonged progression-free survival with acalabrutinib compared with investigator’s choice of idelalisib plus rituximab or bendamustine plus rituximab in patients with relapsed or refractory disease [14]. Likewise, the ECOG-ACRIN E1912 trial demonstrated significantly improved progression-free and overall survival with ibrutinib plus rituximab compared with fludarabine, cyclophosphamide, and rituximab in previously untreated younger patients, while the SEQUOIA trial reported superior progression-free survival (PFS) with zanubrutinib compared with bendamustine plus rituximab in previously untreated patients [15,16]. Additional randomized trials have evaluated BTK inhibitor-based strategies across relapsed or refractory and treatment-naïve populations, although treatment effects vary according to disease setting, comparator regimen, BTK inhibitor, treatment combination, duration of follow-up, and reported clinical outcomes [12,13,17-20].

Despite the growing body of evidence supporting BTK inhibitor-based therapy, important questions remain regarding its comparative efficacy relative to rituximab-containing regimens. Although PFS has generally favored BTK inhibitor-based treatment, the magnitude of benefit for overall survival (OS) remains less certain because of differences in follow-up duration, subsequent therapies, and treatment strategies across trials. Furthermore, the available trials differ substantially in patient populations, treatment settings, comparator regimens, and BTK inhibitor strategies. As additional RCTs have been published in recent years, an updated synthesis of the available evidence is warranted to better characterize the comparative effectiveness of BTK inhibitor-based therapy across these clinically distinct settings [12-20].

Therefore, we conducted a systematic review and meta-analysis of RCTs comparing BTK inhibitor-based therapy with rituximab-containing regimens in patients with CLL, including eligible CLL/small lymphocytic lymphoma (SLL) populations. The primary objectives were to compare PFS and OS between treatment strategies, while the secondary objective was to evaluate overall response rate (ORR) and provide a comprehensive synthesis of the available randomized evidence.

## Review

Materials and methods

Study Design

This systematic review and meta-analysis was conducted and reported in accordance with the Preferred Reporting Items for Systematic Reviews and Meta-Analyses (PRISMA) 2020 statement [21]. The methodology for study identification, study selection, data extraction, risk-of-bias assessment, and quantitative synthesis was conducted in accordance with the recommendations of the Cochrane Handbook for Systematic Reviews of Interventions [22]. The review was restricted to RCTs evaluating BTK inhibitor-based therapy compared with rituximab-based regimens in adult patients with CLL or SLL.

Registration

The review protocol was not prospectively registered. Eligibility criteria, outcome definitions, data-extraction procedures, risk-of-bias assessment, and analytical decisions were therefore reported in sufficient detail to improve transparency and reproducibility.

Literature Search Strategy

A comprehensive electronic literature search was conducted in PubMed/MEDLINE and the Cochrane Central Register of Controlled Trials (CENTRAL) from database inception to August 2, 2026. The search strategy combined controlled vocabulary, including Medical Subject Headings (MeSH), with relevant free-text terms related to CLL, SLL, BTK inhibitors, and rituximab. Search terms included “Tyrosine Kinase Inhibitors,” “Tyrosine Protein Kinase Inhibitors,” “Bruton Tyrosine Kinase,” “BTK inhibitor,” “ibrutinib,” “acalabrutinib,” “zanubrutinib,” “pirtobrutinib,” “LOXO-305,” “Jaypirca,” “Calquence,” “Brukinsa,” “chronic lymphocytic leukemia,” “small lymphocytic lymphoma,” and “rituximab.” Boolean operators (AND/OR) were used to combine the search concepts, and the search strategy was adapted to the syntax and indexing requirements of each database. The electronic search identified 765 records, including 473 records from PubMed/MEDLINE and 292 records from CENTRAL. Reference lists of eligible studies and relevant systematic reviews and meta-analyses were also manually screened; however, no additional eligible records were identified.

Eligibility Criteria

Study eligibility was determined according to the Population, Intervention, Comparator, Outcomes, and Study Design (PICOS) framework [22]. Studies were considered eligible if they were RCTs enrolling adults aged 18 years or older with confirmed CLL or SLL requiring treatment; evaluated a BTK inhibitor-based treatment strategy, including ibrutinib, acalabrutinib, zanubrutinib, or pirtobrutinib, either alone or as part of a combination regimen; compared the BTK inhibitor-based strategy with a rituximab-containing comparator regimen, including rituximab monotherapy, rituximab-containing chemoimmunotherapy, or other treatment combinations containing rituximab; and reported at least 1 of the prespecified efficacy outcomes, namely, PFS, OS, or ORR, with sufficient information for quantitative synthesis. When an otherwise eligible RCT included multiple treatment groups, only participants from the prespecified BTK inhibitor-based versus rituximab-containing comparison were included in the quantitative synthesis; treatment groups that did not meet the predefined eligibility criteria were excluded from the analysis. Studies were excluded if they were observational or nonrandomized studies, single-arm trials, case reports, case series, reviews, editorials, letters without relevant primary trial data, animal studies, duplicate publications, conference abstracts without sufficient outcome data, studies involving an ineligible population, or studies in which rituximab and a BTK inhibitor were administered together without a separate eligible BTK inhibitor-based versus rituximab-containing comparison. When multiple publications originated from the same randomized trial, they were treated as reports of a single study and not as independent trials to avoid double-counting participants.

Study Selection

All retrieved records were imported into Rayyan, a web-based systematic review screening platform, for citation management, deduplication, and study screening [23]. Following removal of duplicate records, two reviewers (HMM and TMK) independently screened titles and abstracts according to the predefined eligibility criteria. Records considered potentially relevant underwent independent full-text assessment by the same reviewers. Disagreements regarding eligibility were resolved through discussion and consensus, and a third reviewer (SY) was consulted when consensus could not be achieved. The study-selection process was documented according to the PRISMA 2020 framework and presented in a PRISMA flow diagram. The search yielded 765 records, of which 158 duplicates were removed, leaving 607 unique records for title and abstract screening. Following title and abstract screening, 52 reports underwent full-text assessment, and 44 were excluded for predefined reasons. Ultimately, eight RCTs were included in the qualitative and quantitative synthesis.

Data Extraction

Data were independently extracted by two reviewers (HMM and TMK) using a standardized data-extraction form developed before data collection. Extracted information included first author, publication year, trial name, study design, study phase, trial registration number where available, treatment setting, sample size, randomization ratio, participant characteristics, intervention regimen, comparator regimen, and duration of follow-up. Efficacy data included PFS, OS, and ORR. For time-to-event outcomes, hazard ratios (HRs) and corresponding 95% confidence intervals (CIs) were extracted for PFS and OS. For ORR, the number of patients achieving an overall response and the total number of patients in each treatment group were extracted. When HRs or corresponding 95% CIs were not directly reported but Kaplan-Meier curves were available, survival data were extracted from the published curves using WebPlotDigitizer [24], and HR estimates were derived using established methods [25]. Where multiple publications were available for the same trial, the publication providing the most complete or most mature efficacy data was preferentially used, with additional reports consulted when required to obtain missing outcome information. For trials containing multiple randomized treatment groups, participant numbers and outcome data were extracted only from the treatment groups meeting the predefined eligibility criteria; therefore, the number of participants contributing to the present meta-analysis could differ from the total number randomized in the original trial. Data extracted from the included trials were independently checked by both reviewers, and discrepancies were resolved through discussion and consensus.

Outcomes

The primary outcomes of this meta-analysis were PFS and OS. PFS was defined according to the individual trial definitions and generally represented the time from randomization to disease progression or death from any cause. OS was defined as the time from randomization to death from any cause. ORR was evaluated as a secondary efficacy outcome and was defined according to the response criteria reported in the individual trials. The quantitative synthesis therefore focused exclusively on PFS, OS, and ORR, consistent with the outcomes available across the included RCTs. All eight included trials contributed PFS data, whereas OS data were available from six trials and ORR data from four trials.

Risk of Bias Assessment

The risk of bias of the included RCTs was independently assessed by two reviewers (HMM and TMK) using the Cochrane Risk of Bias 2 (RoB 2) tool [26]. The assessment considered five domains: bias arising from the randomization process, bias due to deviations from intended interventions, bias due to missing outcome data, bias in measurement of the outcome, and bias in selection of the reported result. Each domain was categorized as having low risk of bias, some concerns, or high risk of bias, and an overall risk-of-bias judgment was assigned according to the RoB 2 framework. Disagreements were resolved through discussion and consensus, with consultation of the third reviewer when necessary. In the final assessment, five of the eight trials were judged to have a low overall risk of bias, whereas three trials raised some concerns. No trial was judged to have a high overall risk of bias.

Statistical Analysis

Statistical analyses were performed using Review Manager (RevMan), version 5.4.1 [27]. Time-to-event outcomes, including PFS and OS, were summarized as HRs with corresponding 95% CIs and pooled using the generic inverse-variance method. An HR below 1.0 favored BTK inhibitor-based therapy. ORR was analyzed as a dichotomous outcome and summarized as risk ratios (RRs) with corresponding 95% CIs using the Mantel-Haenszel method, with an RR greater than 1.0 favoring BTK inhibitor-based therapy. Because substantial clinical and methodological heterogeneity was anticipated across the included trials with respect to treatment setting, BTK inhibitor agent and generation, use of monotherapy or combination therapy, comparator regimen, and duration of follow-up, random-effects models were used for the quantitative analyses. Statistical heterogeneity was assessed using Cochran's Q test and quantified using the I² statistic and between-study variance (Tau²). I² values of approximately 25%, 50%, and 75% were interpreted as representing low, moderate, and high heterogeneity, respectively. A two-sided P-value <0.05 was considered statistically significant.

Sensitivity and Subgroup Analyses

The primary quantitative synthesis was performed using random-effects models because of the clinical and methodological heterogeneity among the included trials. Because of the limited number of eligible RCTs and the small number of studies contributing to individual outcomes, formal subgroup analyses were not performed. Clinically relevant differences in treatment setting and treatment strategy were therefore considered qualitatively when interpreting the pooled estimates. A leave-one-out sensitivity analysis was performed for the PFS outcome using Review Manager (RevMan), version 5.4.1, in which each trial was omitted in turn and the pooled HR and I² statistic were recalculated in order to identify trials contributing disproportionately to the observed heterogeneity.

Assessment of Publication Bias

Publication bias and small-study effects were planned to be assessed by visual inspection of funnel plots when sufficient studies were available. Because fewer than 10 RCTs contributed to each individual pooled outcome, formal statistical assessment of funnel plot asymmetry was not performed, in accordance with recommendations from the Cochrane Handbook. Therefore, the possibility of publication bias or small-study effects could not be reliably excluded.

Ethical Considerations

This systematic review and meta-analysis used data derived exclusively from previously published studies and did not involve direct recruitment, intervention, or interaction with human participants. No individually identifiable participant-level data were collected or accessed by the investigators. Therefore, institutional ethical approval and informed consent were not required for this study. The analysis was conducted in accordance with accepted methodological standards for systematic reviews and meta-analyses, and the findings of the original studies were reported without modification of participant-level information.

Results

Study Selection

The electronic database search identified 765 records, comprising 473 records from PubMed/MEDLINE and 292 records from the Cochrane Central Register of Controlled Trials (CENTRAL). After removal of 158 duplicate records, 607 unique records remained for title and abstract screening. Of these, 555 records were excluded during title and abstract screening. The remaining 52 records underwent full-text assessment. Following full-text evaluation, 44 reports were excluded for predefined reasons, including an incorrect intervention, inappropriate study design, ineligible outcomes, unavailable full-text publication, conference or meeting abstract only, inappropriate publication type, an ineligible population, or foreign-language publication. Ultimately, eight RCTs met the eligibility criteria and were included in both the qualitative and quantitative syntheses. Figure 1 presents the PRISMA 2020 flow diagram illustrating the identification, screening, eligibility assessment, and inclusion of studies in the systematic review and meta-analysis.

**Figure 1:**
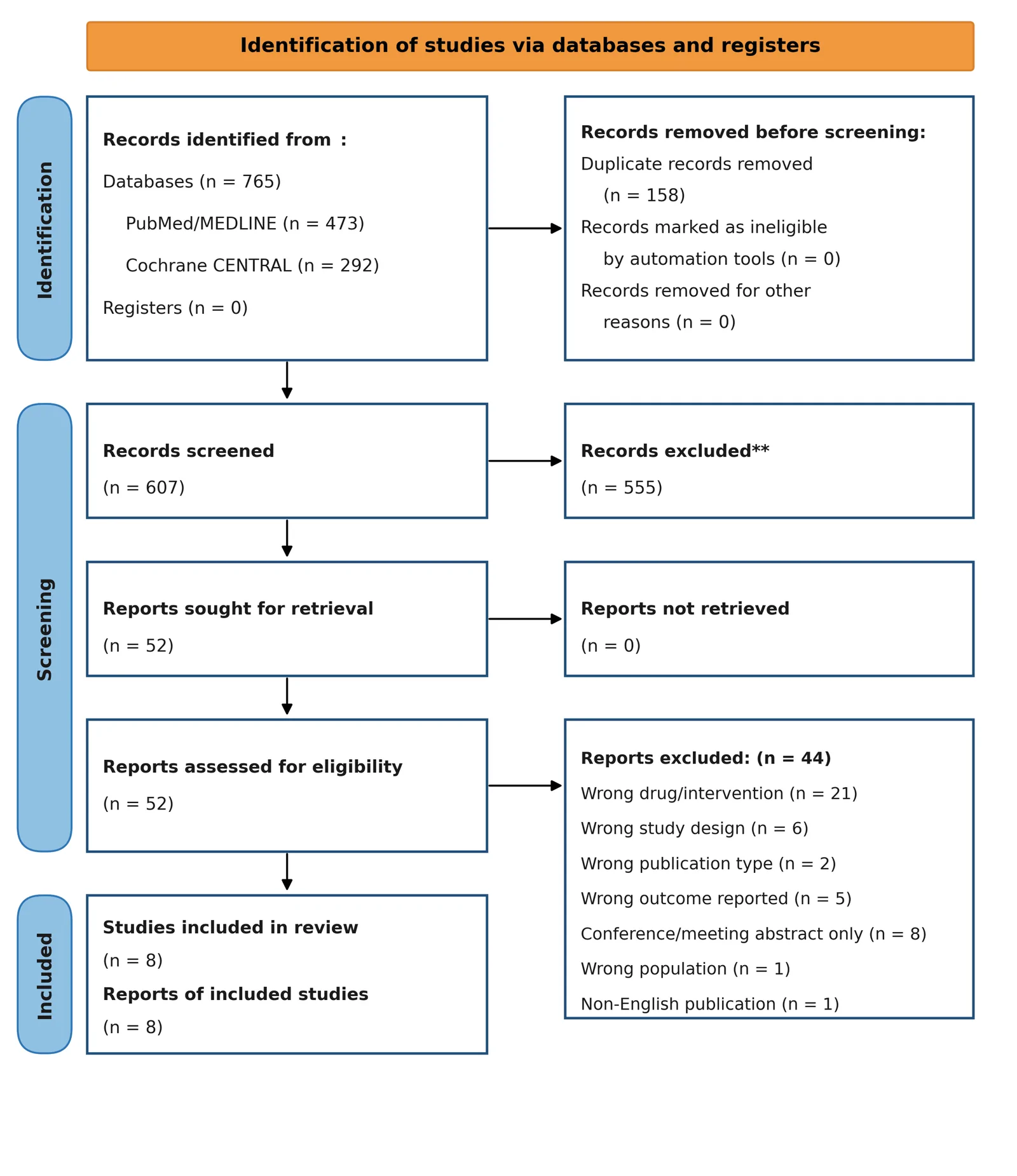
PRISMA 2020 flow diagram illustrating the study selection process and application of the predefined eligibility criteria. PRISMA: Preferred Reporting Items for Systematic Reviews and Meta-Analyses [21]

Characteristics of Included Studies

The eight included RCTs comprised a total of 2,781 participants in the original randomized populations. In trials containing additional randomized treatment groups that did not meet the prespecified eligibility criteria, only participants from the eligible BTK inhibitor-based and rituximab-containing comparison groups were included in the present meta-analysis. The individual trials evaluated several BTK inhibitor-based treatment strategies, including ibrutinib, acalabrutinib, zanubrutinib, and pirtobrutinib, administered either as monotherapy or in combination with other targeted agents. Comparator regimens consisted of rituximab-based treatment strategies, including rituximab monotherapy, rituximab-containing chemoimmunotherapy, or combinations containing rituximab with idelalisib or bendamustine. The included trials enrolled both previously untreated and relapsed or refractory patients with CLL or SLL.

The included studies were GAIA/CLL13 [5], ASCEND [14], the randomized trial by Huang et al. [15], BRUIN CLL-313 [12], the randomized trial by Li et al. [19], ERADIC [20], BRUIN CLL-321 [13], and SEQUOIA [18]. The treatment regimens, comparator therapies, patient characteristics, and follow-up durations varied across the included trials, reflecting differences in disease setting and the evolving use of BTK inhibitor-based treatment in CLL. Table 1 summarizes the key characteristics of the eight RCTs included in the meta-analysis, including study design, patient population, interventions, comparators, and follow-up duration.

**Table 1:** Characteristics of the randomized controlled trials included in the meta-analysis. BTK: Bruton tyrosine kinase; BTKi: Bruton tyrosine kinase inhibitor; CLL: chronic lymphocytic leukemia; SLL: small lymphocytic lymphoma; RCT: randomized controlled trial; I/C: intervention/control; FCR: fludarabine, cyclophosphamide, and rituximab; BR: bendamustine and rituximab; MRD: measurable residual disease; NR: not reported. For trials containing additional randomized treatment groups that did not meet the prespecified eligibility criteria, only the eligible BTK inhibitor-based and rituximab-containing comparison groups were included in the present meta-analysis; therefore, the number of participants analyzed may differ from the total number randomized in the original trial. ¹ The original trial randomized 926 participants; 697 participants from the prespecified eligible comparison groups contributed to the present meta-analysis. ² NR, not reported.

Study (Trial)	Design	N (total)	Sex, M/F	Groups, N (I/C)	Median age, Y (I/C)	Intervention	Comparator
Eichhorst et al., 2023 [5]	RCT	926 (697 in this analysis)^1^	496/201	231/466	61 vs 62	Ibrutinib 420 mg/day + venetoclax, added to obinutuzumab from cycle 1, over 12 cycles	Chemoimmunotherapy: FCR (≥65 y) or BR (>65 y), with rituximab 375 mg/m² (cycle 1) then 500 mg/m² (cycles 2–6)
Ghia et al., 2020 [14]	RCT	310	208/102	155/155	68/67	Acalabrutinib 100 mg twice daily	Idelalisib + rituximab or bendamustine + rituximab (investigator's choice)
Huang et al., 2018 [15]	RCT	160	113/47	106/54	63.6±10.4 (median 65) / 63.6±13.0 (median 67)	Ibrutinib 420 mg once daily until progression/toxicity	Rituximab 375 mg/m² IV once weekly × 4 weeks
Jurczak et al., 2026 [12]	RCT	282	173/109	141/141	65/66	Pirtobrutinib 200 mg once daily until progression/toxicity	Bendamustine 90 mg/m² (d1–2) + rituximab 375→500 mg/m², six 28-day cycles
Li et al., 2026 [19]	RCT	155	90/65	77/78	64/67	Acalabrutinib 100 mg twice daily	Chlorambucil 0.5 mg/kg (d1 & 15) + rituximab 375→500 mg/m², six 28-day cycles
Quinquenel et al., 2026 [20]	RCT	120	88/32	59/61	61/59	Ibrutinib 420 mg/day + venetoclax (ramp-up to 400 mg/day), MRD-guided duration (to month 15 or 27)	Fludarabine 40 mg/m² + cyclophosphamide 250 mg/m² (d2–4) + rituximab 375→500 mg/m², six 4-week cycles (FCR)
Sharman et al., 2025 [13]	RCT	238	166/72	119/119	66/68	Pirtobrutinib 200 mg once daily	Idelalisib + rituximab or bendamustine + rituximab (investigator's choice), in BTKi-pretreated patients
Tam et al. 2022 [18]	RCT	590	377/213	352/238	NR²	Zanubrutinib 160 mg twice daily	Bendamustine 90 mg/m² (d1–2) + rituximab 375→500 mg/m², six cycles

Risk of Bias

The methodological quality of all eight included RCTs was assessed using the Cochrane RoB 2 tool. Five trials were judged to have a low overall risk of bias, whereas three trials raised some concerns. The trials judged to have a low overall risk of bias included GAIA/CLL13 [5], BRUIN CLL-313 [12], ERADIC [20], BRUIN CLL-321 [13], and SEQUOIA [18]. Some concerns were identified for the trials by Ghia et al. [14], Huang et al. [15], and Li et al. [19], primarily related to deviations from intended interventions, outcome measurement, and the randomization process, respectively. No trial was judged to have a high overall risk of bias. All included trials were considered to have a low risk of bias regarding missing outcome data and selection of the reported result. Figures 2, 3 present the risk-of-bias assessment of the RCTs included in the meta-analysis using the Cochrane risk-of-bias tool.

**Figure 2:**
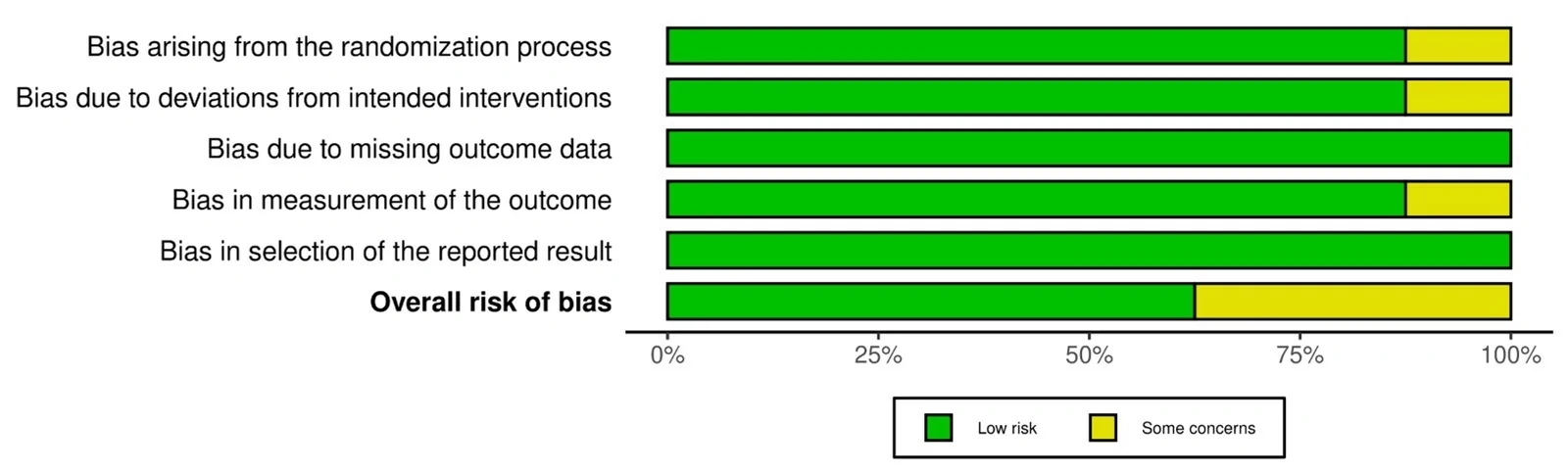
Risk-of-bias summary for the included randomized controlled trials using the Cochrane Risk of Bias 2 (RoB 2) tool.

**Figure 3:**
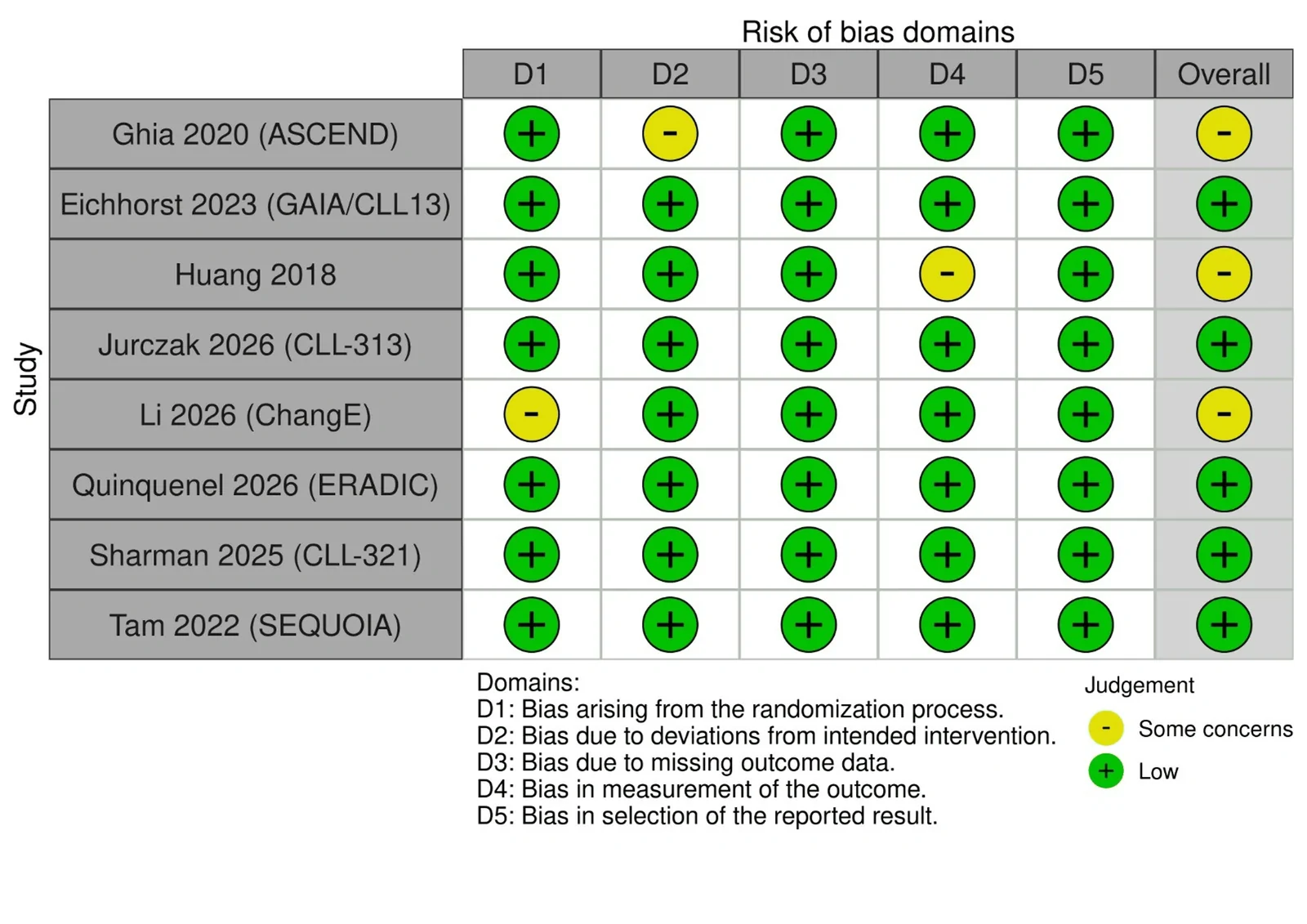
Risk-of-bias judgments across individual domains for the included randomized controlled trials using the Cochrane Risk of Bias 2 (RoB 2) tool. Risk-of-bias assessment of the included randomized controlled trials using the Cochrane Risk of Bias 2 (RoB 2) tool. The included studies are cited as references [5,12,13,14,15,18,19,20].

Progression-Free Survival

All eight included RCTs contributed data to the meta-analysis of PFS. Using a random-effects model, BTK inhibitor-based therapy was associated with a statistically significant reduction in the risk of disease progression or death compared with rituximab-based regimens. The pooled HR was 0.42 (95% confidence interval (CI): 0.24-0.73; Z = 3.08; P = 0.002), corresponding to an approximately 58% relative reduction in the hazard of disease progression or death with BTK inhibitor-based therapy.

Substantial statistical heterogeneity was observed among the included studies (Tau² = 0.55; χ² = 102.45, degrees of freedom = 7; P < 0.00001; I² = 93%). The magnitude of the treatment effect varied across individual trials. The reported HRs ranged from 0.20 (95% CI: 0.11-0.37) in BRUIN CLL-313 [12] to 1.20 (95% CI: 0.98-1.46) in the trial by Huang et al. [15]. Despite this variability, the pooled estimate demonstrated a statistically significant PFS benefit favoring BTK inhibitor-based therapy. Figure 4 presents the forest plot of PFS comparing BTK inhibitor-based therapy with rituximab-based comparator regimens.

**Figure 4:**
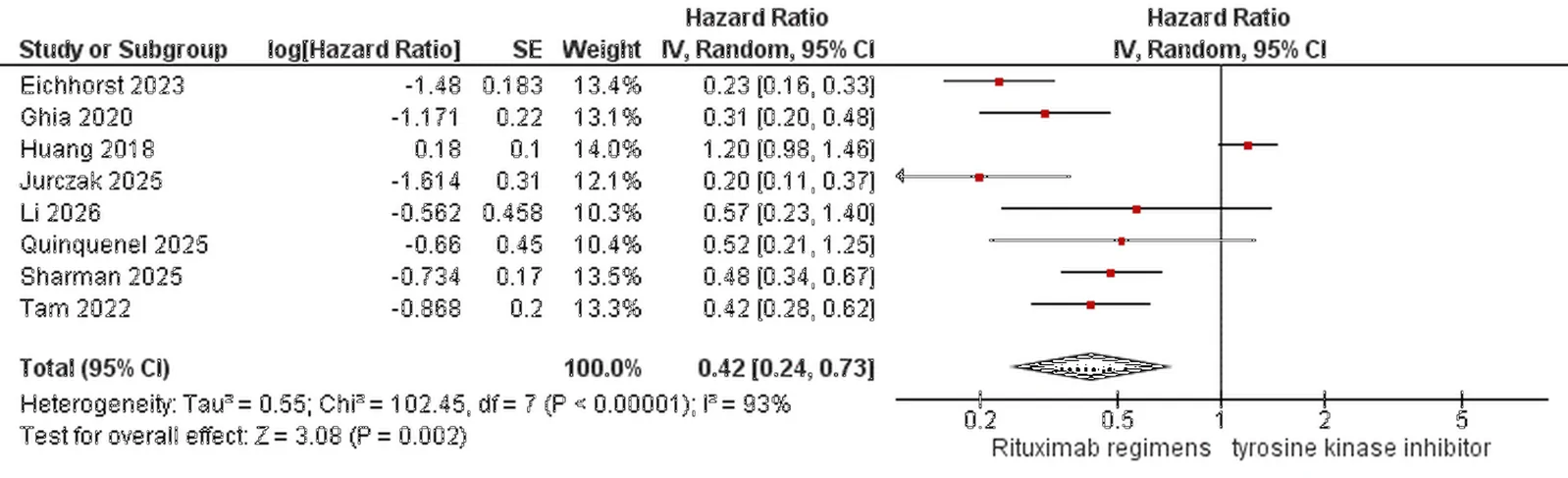
Forest plot of the meta-analysis of progression-free survival comparing BTK inhibitor-based therapy with rituximab-based regimens. BTK: Bruton tyrosine kinase; HR: hazard ratio; CI: confidence interval. The studies included in the forest plot are cited as references [5,12,13,14,15,18,19,20].

Overall Response Rate

Four RCTs contributed data to the meta-analysis of ORR, including ASCEND [14], the trial by Huang et al. [15], BRUIN CLL-313 [12], and the randomized trial by Li et al. [19]. Overall response was achieved in 440 of 543 patients (81.0%) receiving BTK inhibitor-based therapy compared with 350 of 491 patients (71.3%) receiving rituximab-based therapy. The pooled random-effects analysis demonstrated a numerically higher ORR with BTK inhibitor-based therapy; however, the difference did not reach statistical significance (RR: 1.18; 95% CI: 0.98-1.42; Z = 1.75; P = 0.08).

Substantial heterogeneity was observed among the four studies (Tau² = 0.02; χ² = 21.73, degrees of freedom = 3; P < 0.0001; I² = 86%). The largest treatment effect was observed in the trial by Huang et al. [15], whereas the remaining studies demonstrated more modest treatment effects favoring BTK inhibitor-based therapy. The variability in effect estimates contributed to the substantial heterogeneity observed in the pooled analysis. Figure 5 presents the forest plot of overall response rate comparing BTK inhibitor-based therapy with rituximab-based comparator regimens.

**Figure 5:**
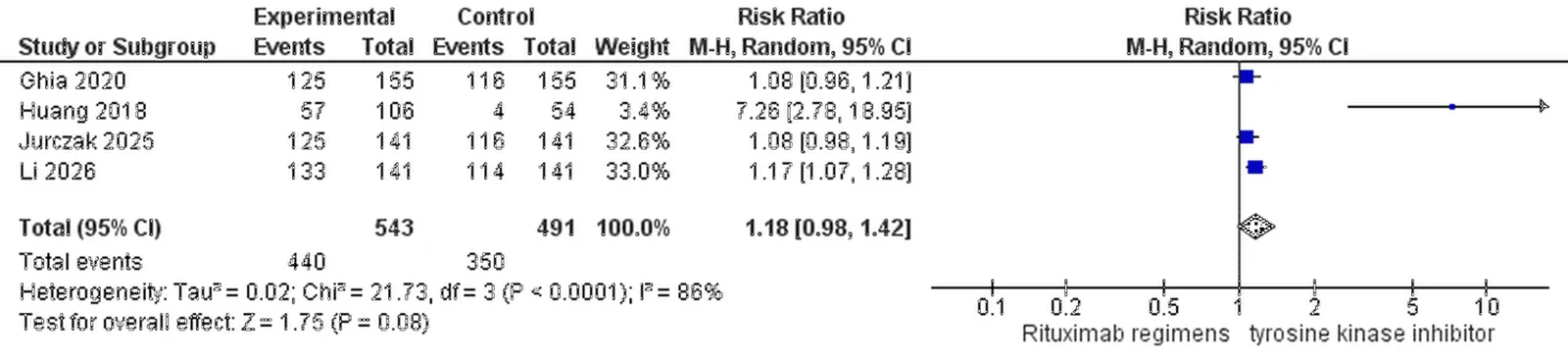
Forest plot of the meta-analysis of overall response rate comparing BTK inhibitor-based therapy with rituximab-based regimens. BTK: Bruton tyrosine kinase; RR: risk ratio; CI: confidence interval. The studies included in the forest plot are cited as references [12,14,15,19].

Overall Survival

Six RCTs contributed data to the OS analysis, including the trials by Huang et al. [15], BRUIN CLL-313 [12], Li et al. [19], ERADIC [20], BRUIN CLL-321 [13], and SEQUOIA [18]. The pooled random-effects analysis demonstrated a numerically lower hazard of death with BTK inhibitor-based therapy compared with rituximab-based regimens; however, this difference was not statistically significant. The pooled HR was 0.67 (95% CI: 0.40-1.11; Z = 1.55; P = 0.12).

Moderate heterogeneity was observed among the six studies (Tau² = 0.16; χ² = 9.64, degrees of freedom = 5; P = 0.09; I² = 48%). Individual study estimates varied across trials, with HRs ranging from 0.26 (95% CI: 0.07-0.94) in BRUIN CLL-313 [2] to 1.09 (95% CI: 0.68-1.75) in BRUIN CLL-321 [13]. Overall, the direction of the pooled effect favored BTK inhibitor-based therapy, although the confidence interval crossed the null value. Figure 6 presents the forest plot of overall survival comparing BTK inhibitor-based therapy with rituximab-based comparator regimens.

**Figure 6:**
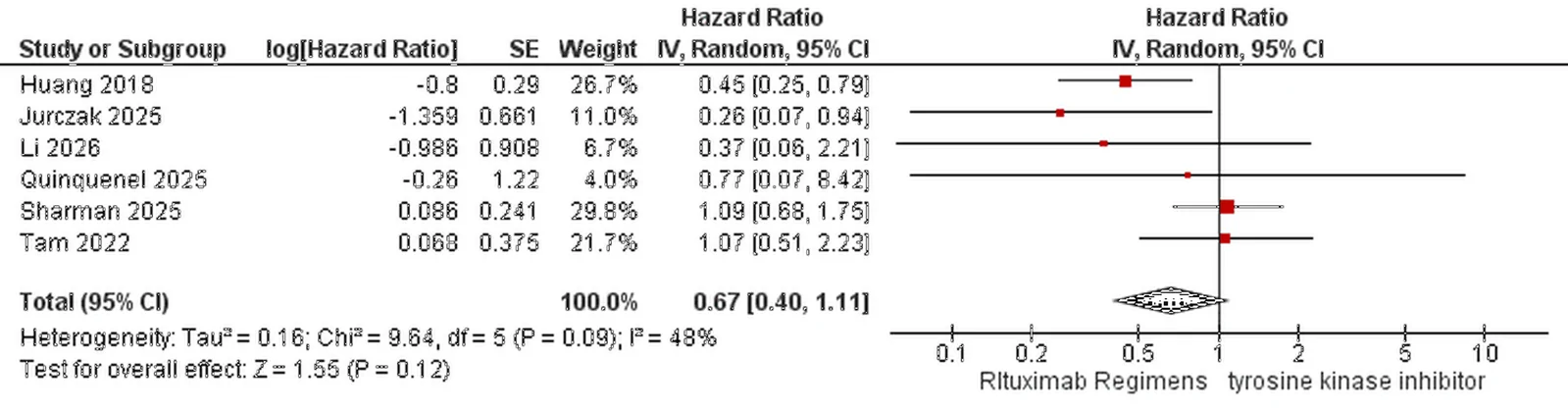
Forest plot of the meta-analysis of overall survival comparing BTK inhibitor-based therapy with rituximab-based regimens. BTK: Bruton tyrosine kinase; HR: hazard ratio; CI: confidence interval. The studies included in the forest plot are cited as references [12,13,15,18,19,20].

Publication Bias

Formal assessment of publication bias was not performed because fewer than 10 RCTs contributed to each individual outcome. Therefore, funnel-plot asymmetry could not be reliably assessed, and the possibility of publication bias or small-study effects could not be excluded.

Subgroup and Sensitivity Analyses

Formal subgroup and sensitivity analyses were not performed because of the limited number of eligible RCTs and the small number of studies contributing to individual outcomes. In particular, only four studies contributed data to the ORR analysis and six studies contributed to the OS analysis, limiting the statistical reliability of additional stratified analyses. The substantial clinical and methodological heterogeneity among the included trials was therefore considered qualitatively in the interpretation of the pooled estimates.

Summary of Findings

Across eight RCTs including 2,781 participants, BTK inhibitor-based therapy demonstrated a statistically significant improvement in PFS compared with rituximab-based regimens, with a pooled HR of 0.42 (95% CI: 0.24-0.73; P = 0.002). ORR was numerically higher with BTK inhibitor-based therapy, although the pooled difference did not reach statistical significance (RR: 1.18; 95% CI: 0.98-1.42; P = 0.08). Similarly, OS numerically favored BTK inhibitor-based therapy, but the pooled difference was not statistically significant (HR: 0.67; 95% CI: 0.40-1.11; P = 0.12). Substantial heterogeneity was observed for PFS and ORR, whereas heterogeneity for OS was moderate. Overall, the findings indicate a clear PFS advantage associated with BTK inhibitor-based therapy, while the available evidence did not demonstrate statistically significant improvements in OS or ORR.

Discussion

In this systematic review and meta-analysis of eight RCTs including 2,781 adults with CLL or SLL, BTK inhibitor-based therapy was associated with significantly longer PFS than the comparator regimens evaluated. The pooled HR was 0.42 (95% CI: 0.24-0.73; P = 0.002), corresponding to an approximately 58% relative reduction in the hazard of progression or death. OS and ORR also numerically favored BTK inhibitor-based therapy but did not reach statistical significance (OS HR: 0.67; 95% CI: 0.40-1.11; P = 0.12; ORR RR: 1.18; 95% CI: 0.98-1.42; P = 0.08). Thus, the most consistent finding was improved disease control rather than a demonstrated survival or response-rate advantage. Contemporary CLL management increasingly emphasizes targeted therapy according to disease biology, treatment setting, comorbidity, and patient preference [1].

Response assessment is standardized by iwCLL criteria, including recognition of treatment-related lymphocytosis that may complicate early response assessment [2]. Rituximab-containing chemoimmunotherapy can produce substantial responses, including with FCR and bendamustine-rituximab, but durable disease control has increasingly favored targeted approaches [3,4]. This may partly explain why the PFS advantage was more apparent than the ORR difference

Interpretation of the PFS result requires caution because heterogeneity was substantial (I² = 93%; Tau² = 0.55). Individual trial estimates ranged from 0.20 (95% CI: 0.11-0.37) in BRUIN CLL-313 to 1.20 (95% CI: 0.98-1.46) in the trial by Huang et al. [12,15]. The random-effects estimate therefore represents an average treatment effect across clinically diverse studies rather than a uniform effect applicable to every patient [22]. The limited number of contributing trials, together with substantial heterogeneity, also reduced the precision and statistical power of the OS and ORR analyses.

The overall direction of the PFS finding is consistent with randomized evidence demonstrating durable disease control with BTK inhibition across several treatment settings in CLL. Ibrutinib improved PFS compared with ofatumumab in previously treated disease [6], while acalabrutinib improved PFS compared with investigator-selected idelalisib plus rituximab or bendamustine plus rituximab in ASCEND [14]. In previously untreated disease, ibrutinib improved disease control compared with chlorambucil, while ibrutinib-based therapy demonstrated superior outcomes compared with chemoimmunotherapy in older patients, and ibrutinib plus rituximab was superior to chemoimmunotherapy in E1912 [7,16,17]. Zanubrutinib also demonstrated superior PFS compared with bendamustine plus rituximab in previously untreated patients in SEQUOIA [18].

These studies provide contextual support for durable disease control with BTK inhibition, although they were not included in the present quantitative comparison. The included studies also reflect the expanding role of newer BTK inhibitors. BRUIN CLL-313 evaluated pirtobrutinib versus bendamustine plus rituximab in previously untreated patients, whereas BRUIN CLL-321 evaluated pirtobrutinib in patients with CLL/SLL previously treated with a covalent BTK inhibitor [12,13]. BTK inhibition disrupts B-cell receptor signaling involved in malignant B-cell survival, proliferation, and microenvironmental interactions, providing a biologically plausible basis for prolonged disease control [28].

The absence of a statistically significant OS benefit should not be interpreted as evidence that BTK inhibitors have no survival effect. Differences in subsequent therapies, crossover, competing mortality, and follow-up duration may attenuate between-group OS differences [29,30]. The pooled estimate remained compatible with both a clinically important reduction and a modest increase in mortality (95% CI: 0.40-1.11). Thus, the evidence is better characterized as inconclusive for OS than as demonstrating no benefit. Longer follow-up may clarify the survival effect [29,31].

ORR was also not significantly different between groups. Rituximab-containing regimens can produce substantial initial responses [3,4], whereas the principal advantage of BTK inhibition may be reflected more strongly in disease-control durability. Treatment-related lymphocytosis may also influence response assessment under iwCLL criteria [2]. Only 4 trials contributed ORR data, with substantial heterogeneity (I² = 86%); therefore, the pooled RR of 1.18 should be interpreted cautiously and should not be considered evidence of equivalent antitumor efficacy.

The marked PFS heterogeneity may reflect differences in treatment setting, prior therapy, intervention, and comparator. Included studies encompassed treatment-naive, relapsed/refractory, and covalent-BTK-inhibitor-pretreated populations [12-15]. Interventions ranged from single-agent BTK inhibitors to time-limited, MRD-guided BTK inhibitor-based combinations, while comparators ranged from rituximab monotherapy to chlorambucil plus rituximab, bendamustine plus rituximab, and fludarabine, cyclophosphamide, and rituximab [12-20]. Duration of follow-up and the maturity of the reported time-to-event data also varied across trials. In a leave-one-out sensitivity analysis, omission of the trial by Huang et al. reduced heterogeneity from I² = 93% to I² = 61%, identifying that trial as the dominant single contributor to between-study variation [15]. It was the only included trial reporting a HR above 1.0 and the only trial employing rituximab monotherapy as the comparator, and it also differed in enrolled population and follow-up duration; its estimate is therefore better regarded as addressing a clinically distinct treatment question than as evidence against a benefit of BTK inhibition. Heterogeneity nonetheless remained substantial after its exclusion, indicating that no single trial accounts for the observed variation. The contrasting estimates from Huang et al. and BRUIN CLL-313 illustrate why the pooled estimate should not be interpreted as a universal class-wide effect [12,15]. With only eight contributing trials, formal subgroup analyses were not considered sufficiently reliable; the potential sources of heterogeneity described above should therefore be regarded as plausible rather than as established treatment-effect modifiers [22].

A class-level result does not imply that individual BTK inhibitors are interchangeable. ELEVATE-RR demonstrated noninferior PFS for acalabrutinib compared with ibrutinib, with fewer cardiovascular adverse events [32,33]. ALPINE similarly demonstrated superior PFS and a favorable cardiovascular safety profile for zanubrutinib compared with ibrutinib [11]. Pirtobrutinib is mechanistically distinct because its noncovalent binding permits activity against BTK C481S-mediated resistance to covalent inhibitors [34,35], with randomized BRUIN CLL-321 data supporting its use after covalent BTK inhibitor exposure [13,35]. Treatment selection should therefore consider prior therapy, disease characteristics, cardiovascular and bleeding risk, comorbidities, and patient preferences [30].

This review has several strengths. Restriction to randomized trials reduced confounding, while duplicate screening, data extraction, and RoB 2 assessment strengthened methodological rigor [21,22,26]. Time-to-event outcomes were synthesized using HRs and inverse-variance methods, with random-effects models accounting for clinical diversity [22,25]. RevMan 5.4.1 was used for quantitative synthesis [27], and summary time-to-event data were reconstructed from Kaplan-Meier curves when necessary using established methods [24,25]. The search was updated through August 2, 2026, incorporating recently reported BTK inhibitor trials [12,19].

Several limitations should be considered. The small number of trials limited statistical power for OS and ORR and the reliability of subgroup and sensitivity analyses. Clinically distinct interventions and patient populations were pooled, contributing to heterogeneity. The review was not prospectively registered, although eligibility and analytical methods were prespecified. Some HRs were reconstructed from digitized Kaplan-Meier curves, introducing uncertainty [24,25]. Most trials were open-label, and publication bias could not be reliably assessed because fewer than 10 studies contributed to outcomes; funnel-plot asymmetry testing has limited reliability with small numbers of studies [22]. Safety, tolerability, and quality-of-life outcomes were outside the scope of this review despite their importance in treatment selection [32,33].

Overall, these findings support BTK inhibitor-based therapy as an effective strategy for prolonging disease control compared with the comparator regimens evaluated. However, substantial heterogeneity and the absence of statistically significant OS or ORR benefits argue against interpreting the pooled estimate as a universal treatment effect. Contemporary recommendations emphasize disease setting, molecular and clinical risk, comorbidities, prior therapy, and patient preference when selecting targeted treatment [1,2,30]. Future research should prioritize head-to-head comparisons of contemporary BTK inhibitors, time-limited versus continuous strategies, MRD-guided approaches, high-risk molecular subgroups, and treatment after covalent BTK inhibitor failure [13,20,35,36]. Longer follow-up and individual participant data meta-analysis may further clarify survival effects and treatment-effect modifiers [22].

## Conclusions

In this meta-analysis of RCTs in patients with CLL/SLL, BTK inhibitor-based therapy was associated with longer PFS than comparator regimens. However, this finding should be interpreted cautiously because heterogeneity was very high, reflecting substantial differences in disease setting, prior therapy, BTK inhibitor, treatment strategy, and comparator regimen. Thus, the pooled HR represents an average across clinically diverse settings rather than a uniform class effect and should not be generalized to every agent, patient population, or treatment context. Leave-one-out analysis identified one trial as the main contributor to heterogeneity, although substantial heterogeneity persisted after its removal. OS and ORR numerically favored BTK inhibitor-based therapy but were not statistically significant and therefore remain inconclusive. Overall, the evidence supports BTK inhibition as an effective disease-control strategy in CLL/SLL, while suggesting that the magnitude of benefit varies by disease setting, prior therapy, inhibitor, and comparator. Treatment selection should therefore remain individualized, with further randomized studies and longer follow-up needed to clarify optimal contemporary and time-limited treatment strategies, safety, quality of life, and patient subgroups most likely to benefit.
